# A Functional Interplay between 5-Lipoxygenase and *μ*-Calpain Affects Survival and Cytokine Profile of Human Jurkat T Lymphocyte Exposed to Simulated Microgravity

**DOI:** 10.1155/2014/782390

**Published:** 2014-09-16

**Authors:** Valeria Gasperi, Cinzia Rapino, Natalia Battista, Monica Bari, Nicolina Mastrangelo, Silvia Angeletti, Enrico Dainese, Mauro Maccarrone

**Affiliations:** ^1^Department of Experimental Medicine & Surgery, Tor Vergata University of Rome, Via Montpellier 1, 00133 Rome, Italy; ^2^Faculty of Veterinary Medicine, University of Teramo, Piazza A. Moro 45, 64100 Teramo, Italy; ^3^StemTeCh Group, Via Colle dell'Ara, 66100 Chieti, Italy; ^4^Faculty of Bioscience, Technology for Food Agriculture and Environment, University of Teramo, Piazza A. Moro 45, 64100 Teramo, Italy; ^5^European Center for Brain Research (CERC), IRCCS Santa Lucia Foundation, Via del Fosso di Fiorano 64-65, 00143 Rome, Italy; ^6^Center of Integrated Research, Campus Bio-Medico University of Rome, Via Alvaro del Portillo 21, 00128 Rome, Italy

## Abstract

A growing body of evidence strongly indicates that both simulated and authentic weightlessness exert a broad range of effects on mammalian tissues and cells, including impairment of immune cell function and increased apoptotic death. We previously reported that microgravity-dependent activation of 5-lipoxygenase (5-LOX) might play a central role in the initiation of apoptosis in human T lymphocytes, suggesting that the upregulation of this enzyme might be (at least in part) responsible for immunodepression observed in astronauts during space flights. Herein, we supplement novel information about the molecular mechanisms underlying microgravity-triggered apoptotic cell death and immune system deregulation, demonstrating that under simulated microgravity human Jurkat T cells increase the content of cytosolic DNA fragments and cytochrome c (typical hallmarks of apoptosis) and have an upregulated expression and activity of *µ*-calpain. These events were paralleled by the unbalance of interleukin- (IL-) 2 and interferon- (INF-) *γ*, anti- and proapoptotic cytokines, respectively, that seemed to be dependent on the functional interplay between 5-LOX and *µ*-calpain. Indeed, we report unprecedented evidence that 5-LOX inhibition reduced apoptotic death, restored the initial IL-2/INF-*γ* ratio, and more importantly reverted *µ*-calpain activation induced by simulated microgravity.

## 1. Introduction

Several studies have shown that authentic space conditions markedly alter physiological processes, thus leading to cardiovascular changes [1], loss of bone density [2, 3], muscle atrophy [2, 4], and immunodepression [5, 6]. To date, it is well established that cells of the immune system are severely affected by microgravity conditions [5–8]. In particular, alterations observed in astronauts and rodents flown in space included altered distribution and function of circulating leukocytes [9–11], lymphocytopenia [12–14], and impaired T cell activation [9, 14–16]. In addition, several* in vivo* and* in vitro* studies reported a weightlessness-dependent alteration of cytokine secretion from T-helper 1 (Th1) and T-helper 2 (Th2) cells that in turn results in a deregulation of cell-to-cell crosstalk as well as of inflammatory responses [9–11, 17].

It has been reported that several proinflammatory Th1 cytokines, including interferon- (INF-) *γ*, tumor necrosis factor- (TNF-) *β* and interleukin- (IL-) 2, and anti-inflammatory Th2 cytokines like IL-4 and IL-10, as well as leukaemia inhibitory factor (LIF), are related to programmed cell death (PCD). These glycoproteins, indeed, are able to induce or protect cells from apoptosis [18–23], so that an alternative classification distinguishes them as anti-(LIF, IL-2, IL-4, IL-10) or proapoptotic (INF-*γ*) substances. A hot topic is the study of the effect of microgravity (be it real or simulated) on apoptosis of different mammalian cell types, including cerebral vascular smooth muscle [24], thyroid cancer [25], endothelial cells [26], cultured glial cells [27], spermatozoa [28], B lymphocytes [29], and T cells [6, 30]. In particular, 5-lipoxygenase (5-LOX) has been proposed as a “gravity responder,” which executes the apoptotic events induced by microgravity in human lymphocytes [6, 30].

Evidence is accumulating that the execution of PCD is finely regulated by a distinct set of signal transduction pathways and catabolic mechanisms (e.g., mitochondrial, lysosomal, and nuclear alterations, lipid modifications, and cytosolic calcium accumulation), and recent data provided first hints that lipid hydroperoxides impact on PCD [31]. Indeed, LOX-catalyzed lipid peroxidation has been reported to be a specific downstream event that triggers apoptosis-inducing factor- (AIF) mediated PCD in primary neurons in culture and in mice [31]. In the same context, calpains cleave multiple substrates potentially involved in PCD and including cyclin-dependent kinase-5 [32], plasma membrane Ca^2+^ ATPase isoform-1 [33], and calcineurin [34]. Also AIF is a calpain substrate implicated in neuronal death, because its proteolysis activates PCD through a translocation of AIF itself from the mitochondria to the nucleus [35, 36].

Against this background, the present study aimed at better defining the influence of the space environment on survival and cytokine profile of human lymphocytes, in order to identify a possible link between these events. In this context, we report an unprecedented functional interplay between 5-LOX and *μ*-calpain in modulating PCD induced by simulated microgravity.

## 2. Materials and Methods

### 2.1. Reagents

Chemicals were of the purest analytical grade. Human recombinants IL-2, IL-4, IL-6, IL-10, INF-*γ*, and LIF, calpain substrate [N-Suc-Leu-Tyr-AMC (7-amido-4-methyl-coumarin)], AA861 (specific inhibitor of 5-LOX), and E64D (specific inhibitor of calpain) were purchased from Sigma Chemical Co. (St. Louis, MO, USA). Mouse anti-cytochrome c antibody was from Cell Signalling Technology Inc. (Danvers, MA, USA); mouse anti-calpain-1 was from Calbiochem (Merck Darmstadt, Germany). Rabbit anti-LIF, anti-IL-2, anti-IL-4, anti-IL-6, anti-IL-10, anti-INF-*γ*, secondary antibodies conjugated to horseradish peroxidase (HRP), and enhanced chemiluminescence (ECL) kit were from Santa Cruz Biotechnology Inc. (Santa Cruz, CA, USA). Goat anti-rabbit conjugated to alkaline phosphatase (GAR-AP) was from Bio-Rad (Hercules, CA, USA).

### 2.2. Simulated Microgravity Cell Cultures

To simulate space conditions, the rotary cell culture system (RCCS), developed by the National Aeronautics and Space Administration (Washington, DC, USA) and manufactured by Synthecon (Houston, TX, USA), was used. Human Jurkat T cells (Clone E6-1) (ATCC, Manassas, VA, USA) were grown in RPMI 1640 medium supplemented with 2 mM glutamine, 2.5 mM sodium pyruvate, 100 U/mL penicillin, 100 *μ*g/mL streptomycin, and 10% heat-inactivated foetal bovine serum. Cells were placed in completely filled 50 mL vessels, to avoid the presence of air bubbles that could lead to shear force damage of cells on the RCCS. Vessels were rotated at a speed of 7.2 rpm (simulated microgravity and referred to as sim-*μ*g), as reported [30, 37], or cultured at ground gravity (1 g), as controls. Incubation of 1 g and sim-*μ*g cells with different compounds was performed at 37°C in an atmosphere of 5% CO_2_, at the indicated concentrations and for the indicated periods of time.

### 2.3. Evaluation of PCD

PCD was estimated by the cell-death detection enzyme-linked immunosorbent assay (ELISA) kit (Boehringer Mannheim, Germany), based on evaluation of histone-associated DNA fragments in the cytoplasm, as previously reported [30].

Cytochrome c release from mitochondria was analyzed as reported [38]. Briefly, cells were lysed in HB buffer (5 mM Tris-HCl pH 7.4, 10 mM KCl, 1 mM MgCl_2_, and 1 mM DTT), containing protease inhibitor cocktail, and centrifuged at 1000 ×g for 10 min to completely remove nuclei and whole cells. The resulting supernatant was centrifuged at 3000 ×g for 10 min; then the pellet was saved as membrane-bound organellar fraction enriched with mitochondria, while the supernatant, after centrifugation at 100000 ×g for 40 min, was collected as cytosolic fraction. These two fractions were analyzed for cytochrome c localization by means of ELISA: mitochondrial and cytosolic proteins (20 *μ*g/well) were incubated with anti-cytochrome c antibody (diluted 1 : 500), and after incubation with a GAR-AP (diluted 1 : 2000), colour development of the alkaline phosphatase reaction was measured at 405 nm (A_405 nm_), by using *p*-nitrophenyl phosphate as substrate.

### 2.4. Analysis of *μ*-Calpain Activity and Expression

Detection of *μ*-calpain mRNA was performed by quantitative reverse transcriptase-polymerase chain reaction (q-RT-PCR), as previously reported [6]. Briefly, total RNA was extracted from Jurkat cells using the RNeasy extraction kit (Qiagen, Crawley, UK), following the manufacturer's instructions. RT-PCR reactions were performed using the RT-PCR SuperScript III Platinum Two-Step qRT-PCR Kit (Invitrogen, Carlsbad, CA, USA). One *μ*g total RNA was used to synthesize cDNA with 10 U/*μ*L SuperScript III reverse transcriptase, in the presence of 2 U/*μ*L RNaseOUT, 1.25 *μ*M oligo (dT), 1.25 ng/*μ*L random hexamers, 5 mM MgCl_2_, 0.5 mM dNTP mix, and DEPC-treated water. The reaction was performed using the following RT-PCR program: 25°C for 10 min, 42°C for 50 min, 85°C for 5 min, and then, after addition of 0.1 U/*μ*L of* E. coli* RNase H, the product was incubated at 37°C for 20 min. For expression studies, target transcripts were amplified in ABI PRISM 7700 sequence detector system (Applied Biosystems, Foster City, CA, USA). Thermal cycling involved 40 cycles of 95°C for 15 sec and 60°C for 30 sec, after initial denaturation for 10 min at 95°C. TaqMan MGB probe was synthesized by Applied Biosystems (Foster City, CA, USA). The probe was labelled with the fluorescent dye 6-carboxyfluorescein at the 5′ end and a dark quencher at the 3′ end (Applied Biosystems). Fluorescence was measured after each cycle of PCR and, to confirm the quality of isolated RNA and to standardize the amount of RNA applied, glyceraldehyde-3-phosphate dehydrogenase (GAPDH) was used as endogenous control with FAMTM dye label and MGB. Real-time PCR mixtures contained template cDNA, 20x Primer/Probe Mix, TaqMan MGB Probe with FAMTM dye label, no primer limitation, Minor Groove Binder and Nonfluorescent Quencher, Universal PCR Master Mix, no AmpErase UNG Applied Biosystems (Foster City, CA, USA) in a total volume of 25 *μ*L in a 96-well plate. Relative *μ*-calpain expression levels were measured by ΔΔCT method (PE-Applied Biosystems; Sequence Detector User Bulletin).

Calpain protein expression was evaluated by Western blot analysis. Briefly, cell lysates (20 *μ*g/well) were subjected to SDS-PAGE, electroblotted onto PVDF membranes, incubated with mouse anti-*μ*-calpain antibody (1 : 4000), which detects both the full-length (large subunit) and the autoproteolytically cleaved forms of *μ*-calpain, and detected with ECL. Calpain quantification was also evaluated through ELISA method, by incubating protein lysates (20 *μ*g/well) with mouse anti-*μ*-calpain-1 (1 : 2000) as primary antibody and HRP-conjugated antibody (1 : 5000) as secondary antibody. The HRP enzymatic activity was determined by adding 100 *μ*L/well of tetramethylbenzidine containing 0.002% H_2_O_2_, and the absorbance was read on a microplate reader (ELISA Ascent Software per Multiskan) at 450 nm. Absorbance values of the samples were within the linearity range of the ELISA test, assessed by calibration curves with known amounts of *μ*-calpain (in the range of 7.5–60.0 ng/well).

The enzymatic activity of *μ*-calpain was measured as reported [39]. Briefly, cell lysates (40 *μ*g/test) were incubated with 150 *μ*M calpain substrate (N-Suc-Leu-Tyr-AMC) in 10 mM Hepes, pH 7.4, 1% Triton X-100, and 100 *μ*M CaCl_2_, for 2 hours at 37°C. After incubation, hydrolyzed AMC groups were measured on a fluorimeter LS50B (Perkin-Elmer Life Sciences Inc., Boston, MA, USA) with an excitation filter of 380 nm and emission filter of 460 nm.

### 2.5. 5-LOX Activity

The activity of 5-LOX (arachidonate:oxygen 5-oxidoreductase; E.C. 1.13.11.34) was determined as previously reported [6]. Briefly, the end product leukotriene (LT) B_4_ was extracted from Jurkat cells (5 × 10^6^ cell/test) and quantified at 405 nm by using the Leukotriene B_4_ EIA Kit (Cayman Chemical Company, Ann Arbor, MA, USA) and calibration curves drawn according to the customer's instructions.

### 2.6. Cytokine Profile Analysis

Jurkat cells harvested after 48 hours were centrifuged at 200 ×g for 10 min to collect cells and culture medium. Cells were lysed in 50 mM Tris-HCl (pH 7.4), containing protease inhibitors, and cytokine content was quantified by coating proteins (20 *μ*g/well) from whole lysates overnight in a 96-well ELISA microplate, as reported [40]. Rabbit anti-LIF, anti-IL-2, anti-IL-4, anti-IL-6, anti-IL-10, and anti-INF*γ* (diluted 1 : 500) were used as primary antibodies; GAR-AP (diluted 1 : 2000) was used as secondary antibody and absorbance values were read at 405 nm. Release of LIF and other cytokines from Jurkat cells into the medium was quantified through Quantikine Immunoassay kit (R&D System, Minneapolis, MN, USA) and a specific Multiprotein Profiling ELISA Kit (SuperArray Bioscience Co., Germany), respectively, according to the manufacturer's instructions. To this aim, 50 *μ*L of culture medium was used, and the content of each protein was evaluated by comparing A_405 nm_ values to those of antigen standard curves (positive controls).

### 2.7. Statistical Analysis

All values were expressed as means ± SEM of at least three independent experiments. Student's unpaired *t*-test or one-way ANOVA (followed by Bonferroni* post hoc* analysis) was used to compare quantitative data with normal distributions and equal variance. The statistical InStat 3 program (GraphPAD Software for Science, San Diego, California) was used, and a value of *P* < 0.05 was considered statistically significant.

## 3. Results

### 3.1. Prolonged Exposure to Simulated Microgravity Induces Apoptosis in Human Jurkat T Cells

Jurkat T cells were exposed to simulated microgravity for different times (from 0 to 48 hours) and the hallmarks of apoptosis DNA fragmentation and cytochrome c release were analyzed. In agreement with previously reported data [30], RCCS treatment led to a time-dependent increase of cytosolic DNA fragments that were undetectable after a brief exposure (4 hours) to simulated microgravity, increased after 24 hours (~2-fold over 1 g cells), and reached a maximum level of ~3-fold over controls 24 hours later (Table 1). Then, the subcellular localization of cytochrome c upon simulated microgravity was checked. Jurkat cells exposed to weightlessness showed a loss of mitochondrial cytochrome c and a parallel increase in the cytosolic content, with a time-dependence comparable to that observed for DNA fragmentation (Table 1). Conversely, Jurkat cells incubated at 1 g under the same experimental conditions did not show significant signs of PCD (Table 1). Since RCCS treatment for 48 hours yielded a significant increase in PCD, we chose to perform all subsequent experiments using this time point.

### 3.2. Prolonged Exposure to Simulated Microgravity Upregulates *μ*-Calpain Expression and Activity in Human Jurkat T Cells

**Figure 1:**
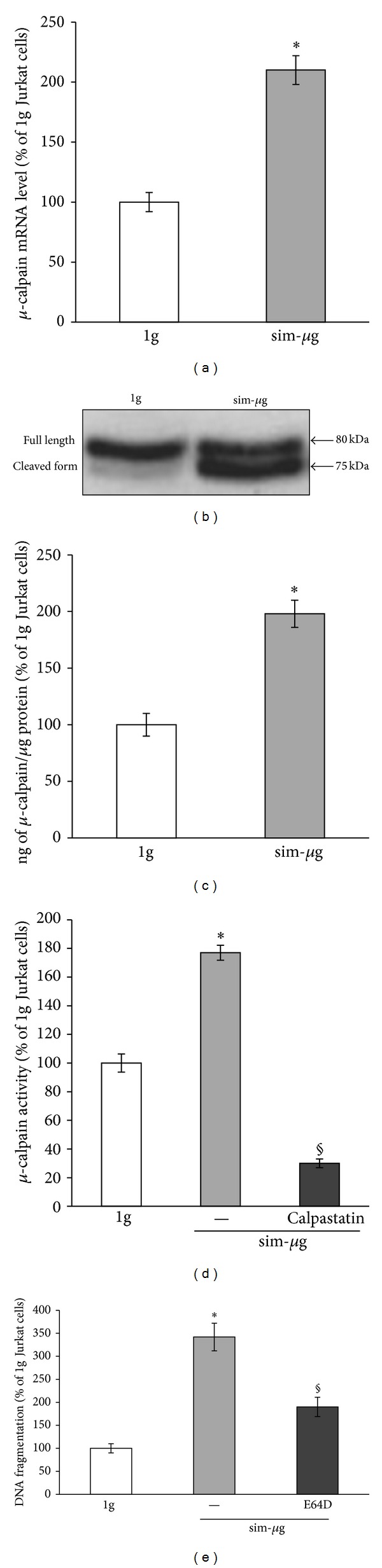
Effect of simulated microgravity on *μ*-calpain expression and activity in Jurkat T cells. (a) RT-qPCR analysis of *μ* *-calpain *gene expression in Jurkat cells exposed to simulated microgravity (sim-*μ*g) at 37°C for 48 hours. Gene levels were normalized to the housekeeping GAPDH and expressed as percentage of 1 g cells considered as control, set to 100 (b). Western blot analysis of protein expression in Jurkat cells treated as in (a). (c) ELISA analysis of *μ*-calpain protein content in Jurkat cells treated as in (a). Results are expressed as percentage of 1 g cells considered as control, set to 100 (= 9.48 ± 0.50 ng/per *μ*g protein) (d) *μ*-calpain activity analysis in Jurkat cells treated as in (a) in absence (−) or in presence of 5 *μ*M of calpastatin. Results are expressed as percentage of 1 g cells considered as control, set to 100 (= 66.26 ± 3.65 pmol/min per mg protein). (e) DNA fragmentation in Jurkat cells exposed to simulated microgravity for 48 hours in absence (−) or in presence of 10 *μ*M E64D. Values are expressed as percentage of 1 g cells considered as control.  *denotes  *P* < 0.001 versus 1 g cells;  ^§^denotes *P* < 0.05 versus sim-*μ*g cells.

We have previously reported that after 48 hours of exposure to authentic microgravity, human lymphocytes show increased mRNA levels of *μ*-calpain [6], a Ca^2+^-dependent intracellular cysteine protease that is implicated in different physiological functions, including cell growth and apoptosis [41]. Therefore, once established that under our experimental conditions Jurkat cells underwent apoptosis, we checked whether RCCS treatment might engage *μ*-calpain. In agreement with our previous data [6], RT-qPCR experiments demonstrated a significant increase of *μ*-calpain mRNA in sim-*μ*g Jurkat cells (~2-fold over 1 g cells) (Figure 1(a)). Interestingly, upregulation of* capn* 1 gene, which encodes *μ*-calpain, was paralleled by increased protein content (Figures 1(b) and 1(c)). Western blot analysis, indeed, showed that 48 hours of RCCS treatment dramatically increased *μ*-calpain protein levels; in particular, larger amounts of autocleaved and active fragment of *μ*-calpain (~75 kDa) [42] were found in sim-*μ*g, whereas no active enzyme was observed in 1 g cells (Figure 1(b)). Such a result was further corroborated by ELISA, revealing that RCCS almost doubled *μ*-calpain protein content after 48 hours (Figure 1(c)). We next determined whether increased mRNA and protein content might result in increased enzyme activity. By analysing the cleavage of a fluorogenic *μ*-calpain substrate, we observed an enhanced protease activity in sim-*μ*g T cells (~2-fold over 1 g cells) (Figure 1(d)). Specific proteolytic activity of calpain was confirmed by the addition of 5 *μ*M calpastatin (Figure 1(d)), the natural calpain inhibitor [43]. Since calpain activation seemed to be implicated in DNA fragmentation [44, 45], we analyzed the effect of E64D, a cell permeable and selective inhibitor of the same protease [46], on simulated microgravity-induced PCD. As shown in Figure 1(e), inhibition of calpain activity significantly lowered internucleosomal DNA fragmentation, thus preventing weightlessness-induced cell death of T cells.

### 3.3. Prolonged Exposure to Simulated Microgravity Affects the Balance between Proapoptotic and Antiapoptotic Cytokines in Jurkat Cells

**Figure 2:**
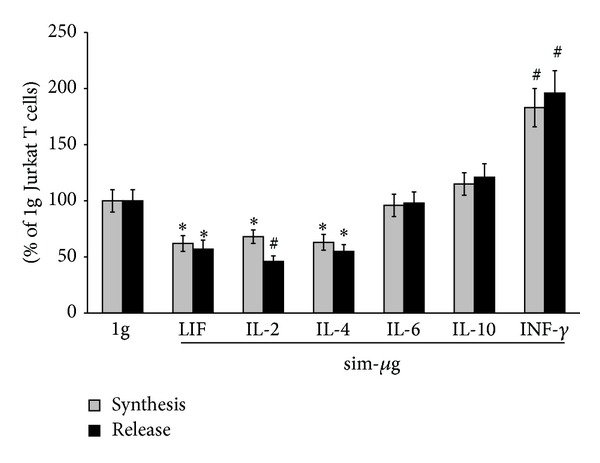
Effect of simulated microgravity on cytokine profile of Jurkat T cells. Cells were exposed (sim-*μ*g) or not exposed (1 g) to simulated microgravity at 37°C for 48 hours and cytokine synthesis (gray bars) and release (black bars) were analyzed as reported in Section 2.6. Results are reported as percentage of 1 g cells set to 100. For synthesis, 100% of IL-2 = 0.27 ± 0.01  A_405 nm_; 100% of IL-4 = 0.34 ± 0.02  A_405 nm_; 100% of LIF = 0.22 ± 0.02  A_405 nm_; 100% of IL-6 = 0.18 ± 0.01  A_405 nm_; 100% of IL-10 = 0.34 ± 0.02  A_405 nm_; 100% of INF-*γ*  0.25 ± 0.02  A_405 nm_. For release, 100% of LIF = 0.42 ± 0.03  Abs_405 nm_; 100% of IL-2 = 6.6 ± 0.5 pg/mL; 100% of IL-4 = 1.2 ± 0.1 pg/mL; 100% of IL-6 = 20 ± 2 pg/mL; 100% of IL-10 = 2.5 ± 0.3 pg/mL; 100% INF-*γ* = 12.2 ± 0.1 pg/mL.  *denotes *P* < 0.05 versus 1 g cells;  ^#^denotes *P* < 0.01 versus 1 g cells.

Then, we characterized the cytokine profile in Jurkat T cells exposed to simulated microgravity. As demonstrated by ELISA assay, 48 hours of RCCS treatment significantly reduced the synthesis and release of antiapoptotic cytokines like LIF, IL-4, and IL-2, while increasing protein levels of the proapoptotic cytokine INF-*γ* (Figure 2). Instead, no change in IL-6 and IL-10 content was observed upon simulated microgravity treatment (Figure 2).

**Figure 3:**
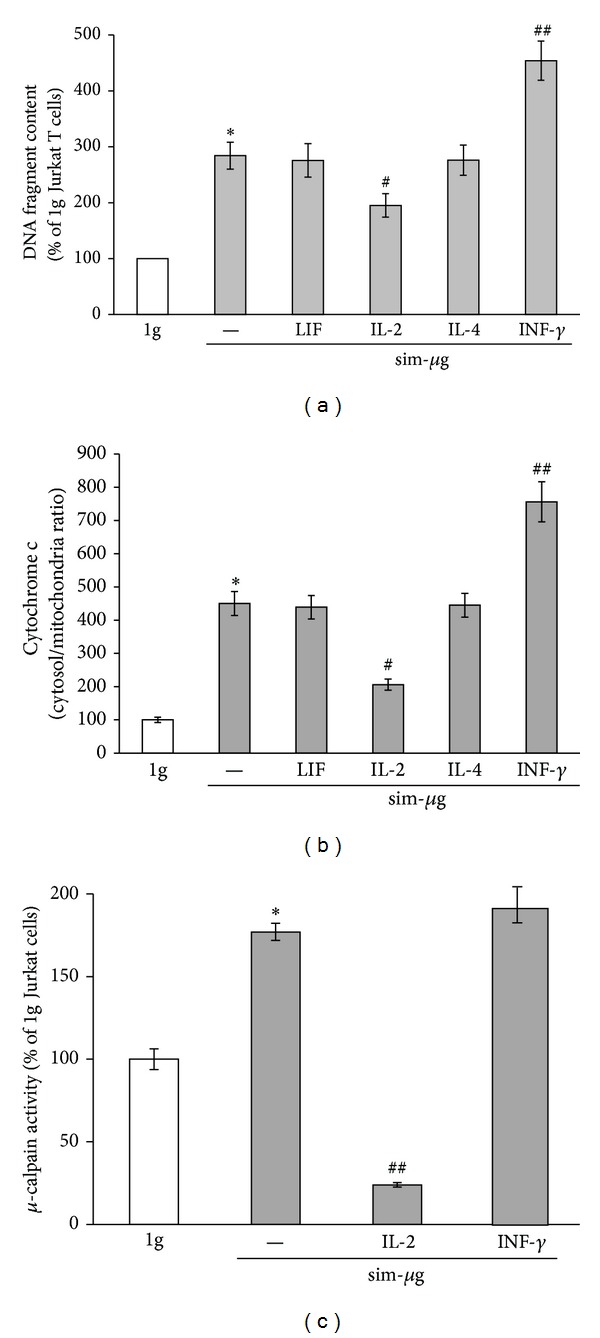
Cytokine effects on Jurkat cell apoptosis under simulated microgravity conditions. Jurkat T cells were exposed (sim-*μ*g) or not exposed (1 g) to simulated microgravity in absence (−) or in presence of the indicated cytokines (10 ng/mL), and DNA fragmentation (a), cytochrome c release (b), and *μ*-calpain activity (c) were evaluated as reported in Section 2. Results are reported as percentage of 1 g cells set to 100. For DNA fragmentation, 100% = 0.300 ± 0.030  A_405 nm_; for cytochrome c release, 100% = 0.074 ± 0.005; for *μ*-calpain activity, 100% = 66.26 ± 3.65 pmol/min per mg protein.  *denotes *P* < 0.001 versus1 g cells;  ^#^denotes *P* < 0.05 versus sim-*μ*g cells;  ^##^denotes *P* < 0.01 versus sim-*μ*g cells.

Next, we went further by investigating whether RCCS-induced PCD might be related to the unbalance between proapoptotic and antiapoptotic cytokines. To this aim, we analyzed apoptosis in Jurkat cells cultured under simulated microgravity for 48 hours, in the presence of the cytokines that changed upon RCCS exposure. Neither LIF nor IL-4 (both at 10 ng/mL) reduced cytosolic DNA fragments (Figure 3(a)) and cytochrome c content (Figure 3(b)); on the other hand, 10 ng/mL IL-2 was able to protect Jurkat cells from simulated microgravity-triggered cell death, since it significantly reduced both DNA fragmentation and cytochrome c release (Figures 3(a) and 3(b)). To validate our hypothesis, we also analyzed the effect of INF-*γ* (10 ng/mL). In agreement with the previous data (Figure 2), the latter cytokine drastically enhanced RCCS-induced PCD of Jurkat cells (~ 4.5- and 2.5-fold over 1 g cells and sim-*μ*g cells, resp.) (Figures 3(a) and 3(b)).

To gain further insights on the evaluation of a possible relationship between altered IL-2/INF-*γ* content and calpain activation, we measured the activity of the latter enzyme in the presence of these two cytokines. Interestingly, IL-2 reduced calpain activation due to simulated microgravity, while INF-*γ* did not significantly affect enzyme activity (Figure 3(c)).

### 3.4. Effect of Inhibition of *μ*-Calpain and 5-LOX on Apoptosis and Cytokine Release

Since we observed that simulated microgravity triggers apoptosis by altering the content of IL-2 and INF-*γ*, we asked whether such an event might engage 5-LOX, which has been proposed as a “gravity responder” [30]. First, we analyzed 5-LOX activity by quantifying the content of its LTB_4_ product upon RCCS exposure. In agreement with previous data, we found an early increase of 5-LOX activity (~2 fold over 1 g cells), with values that remained unchanged over the whole time period tested (Table 2). Hence, we subjected Jurkat cells to simulated microgravity in the presence of 10 *μ*M AA861, a specific 5-LOX inhibitor [47]. As shown in Table 3, we observed that 5-LOX inhibition reduced DNA fragmentation and cytochrome c release and reverted calpain activation. More interestingly, it was able to restore the balance between IL-2/and INF-*γ* that was altered by RCCS treatment. These data seem to suggest that increased 5-LOX activity might be (at least in part) responsible for altered cytokine levels.

## 4. Discussion

The effects of LTs on the secretion of cytokines have been reported both* in vitro* and* in vivo* [46]. Here, we demonstrated that increased LTB_4_ synthesis upon simulated microgravity exposure is paralleled by a reduced release of antiapoptotic cytokines, such as LIF, IL-4, and IL-2 [19–23], as well as by a significant increase of the production of the proapoptotic cytokine INF-*γ* [18, 23]. These data are in line with the immunomodulatory role postulated for 5-LOX metabolites, and especially for LTB_4_. Indeed, the latter substance is a powerful chemoattractant for inflammatory cells and induces degranulation, superoxide anion production, and adherence of neutrophils to vascular endothelial cells [48]. LTB_4_ has been already demonstrated to affect the production of several cytokines, including IL-1*β* [49, 50], IL-2 [51, 52], IL-6 [53], INF-*γ* [54], IL-4 [55], and IL-10 [56]. Moreover, LTB_4_ has been also demonstrated to modulate the expression of the IL-2 receptor *β*-chain in natural killer cells and in CD8^+^ lymphocytes [57].

In this context, our data add further information on the mechanism of PCD activation, suggesting a crosstalk between 5-LOX and *μ*-calpain signalling. In particular, we demonstrate that exposure of Jurkat T cells to simulated microgravity induced activation of *μ*-calpain and 5-LOX. Our results suggest that the functional interplay between these two enzymes could be related to the synthesis of a specific pattern of cytokines. In line with this, our results show that 5-LOX inhibition (i) reduced DNA fragmentation and cytochrome c release (typical apoptotic markers); (ii) reestablished the initial IL-2/INF-*γ* ratio; and (iii) more importantly reverted *μ*-calpain activation induced by simulated microgravity (Table 3). Furthermore, we showed that treatment of Jurkat T cell with IL-2, whose levels are downregulated upon simulated microgravity exposure (Figure 2), significantly reduced *μ*-calpain activation upon RCCS treatment. Remarkably, the latter result is in agreement with the well-known antiapoptotic effect of IL-2 [21, 23]. It should be noted that the lack of any increase in *μ*-calpain activity in the presence of 5-LOX inhibitors might be suggestive that additional and as-yet-unknown 5-LOX products are able to directly activate *μ*-calpain. Thus, in addition to a specific role of distinct cytokines in modulating the crosstalk between 5-LOX and *μ*-calpain, we can speculate that 5-LOX activation could also induce the formation of specific lipid hydroperoxides that could trigger PCD* via* *μ*-calpain activation. In line with the latter hypothesis, hydroperoxides of cardiolipin and phosphatidylserine have been detected as byproducts upon PCD [58]. Consistently, it has been demonstrated that LOX-induced lipid peroxidation triggers AIF-mediated PCD [31]. Indeed, although a finely regulated lipid peroxidation may have beneficial effects for the cells and the whole organism, leading to different physiological roles of LOXs (such as eicosanoid synthesis, cell maturation, and lipid mobilization), when the lipid bilayer of biological membranes is oxidized in an uncontrolled manner (as in the case of external stimuli like microgravity), it may lose its barrier function and thus harm the integrity of subcellular organelles or of the whole cell [59]. Consistently, an overactivated 5-LOX can open pore-like structures in mitochondrial membranes [60, 61], thus forming the basis for a converging role of this enzyme in the induction of PCD by unrelated stimuli [59].

Overall, our results demonstrate that simulated microgravity-dependent increase in 5-LOX activity regulates survival and cytokine release of human T lymphocytes by engaging *μ*-calpain.

## 5. Conclusions

Our findings seem to add biochemical support to the immunodepression observed in astronauts exposed to authentic microgravity for long periods of time (e.g., International Space Station crew members or astronauts travelling to Mars). Taking into account that Jurkat E6.1 cells are somewhat different from normal human T cells [61], nonetheless, they are considered a valid experimental model, especially in the light of their exaggerated signaling, making changes much easier to detect. Therefore, only authentic space conditions will give a conclusive answer on whether or not the unbalance between proapoptotic and antiapoptotic cytokines due to impaired 5-LOX and *μ*-calpain activities can affect immune response, helping to design countermeasures against apoptosis observed in space.

## Figures and Tables

**Table 1:** Time-dependent effect of simulated microgravity on apoptotic markers in Jurkat T cells exposed to simulated microgravity (sim-*μ*g) or kept at normal gravity (1g).

Parameter	1g	4-hour sim-*μ*g	24-hour sim-*μ*g	48-hour sim-*μ*g
DNA fragmentation	100 ± 8	107 ± 8	270 ±18*	342 ±21**
Cytochrome c release (cytosol/mitochondria ratio)	100 ± 9	111 ± 7	347 ±41*	450 ±47**

Results are expressed as percentage of 1g cells set to 100. For DNA fragmentation, 100% = 0.300 ± 0.030 A_405 nm_; for cytochrome c release, 100% = 0.074 ± 0.005. *denotes *P*< 0.01 versus 1g cells; **denotes *P*< 0.001 versus 1g cells.

**Table 2:** Time-dependent effect of simulated microgravity on 5-LOX activity in Jurkat cells.

Sample	LOX activity (% of 1g cells)^a^
1g cells	100 ± 11
4-hour sim-*μ*g cells	213 ± 18*
24-hour sim-*μ*g cells	249 ± 23*
48-hour sim-*μ*g cells	235 ± 21*

^a^100% of 5-LOX activity = 89 ± 7 pg of LTB_4_/1 × 10^6^ cells. *denotes *P* < 0.01 versus 1g cells.

**Table 3:** Effect of 5-LOX inhibition on Jurkat T cells exposed for 48 hours to simulated microgravity (sim-*μ*g) or kept at normal gravity (1g).

Parameter	1g	sim-*μ*g	sim-*μ*g + 10 *μ*M AA861
DNA fragmentation	100 ± 10	342 ± 21***	250 ± 11^#^
Cytochrome c release (cytosol/mitochondria ratio)	100 ± 9	450 ± 47***	230 ± 24^#^
Calpain activity	100 ± 11	177 ± 9***	31 ± 2^#^
IL-2 protein content	100 ± 9	67 ± 5*	93 ± 8^#^
INF-*γ* protein content	100 ± 9	179 ± 15**	120 ± 4^#^

Values are reported as percentage of relative control set to 100. For DNA fragmentation, 100% = 0.30 ± 0.03 A_405 nm_; for cytochrome c release, 100% = 0.074 ± 0.005; for calpain activity, 100% = 66.26 ± 3.65 pmol/min per mg protein; for IL-2 synthesis, 100% = 0.27 ± 0.01 A_405 nm_; for INF-*γ* synthesis, 100% = 0.25 ± 0.02 A_405 nm_. *denotes = *P* < 0.05 versus 1g cells; **denotes *P* < 0.01 versus 1g cells; ***denotes *P* < 0.001 versus 1g cells; ^#^denotes *P* < 0.01 versus sim-*μ*g cells.

## Abbreviations

LOX: Lipoxygenase
AMC: 7-Amido-4-methyl-coumarin
AIF: Apoptosis-inducing factor
RCCS: Rotary cell culture system
ECL: Enhanced chemiluminescence
ELISA: Enzyme-linked immunosorbent assay
GAPDH: Glyceraldehyde-3-phosphate dehydrogenase
GAR-AP: Goat anti-rabbit conjugated to alkaline phosphatase
1 g: Ground gravity
HRP: Horseradish peroxidase
INF-*γ*: Interferon-*γ*
IL-2: Interleukin-2
LIF: Leukaemia inhibitory factor
(LT)B_4_: Leukotriene B_4_
sim-*μ*g: Simulated microgravity
PCD: Programmed cell death
q-RT-PCR: Quantitative reverse transcriptase-polymerase chain reaction
Th1: T-helper 1
Th2: T-helper 2
TNF-*β*: Tumor necrosis factor-*β*.
